# Dietary Oxidized Linoleic Acid Modulates Plasma Lipids beyond Triglycerides Metabolism

**DOI:** 10.1155/2017/1645828

**Published:** 2017-11-29

**Authors:** Mahdi Garelnabi, Gregory Ainsworth, Halleh Mahini, Naseeha Jamil, Chinedu Ochin

**Affiliations:** ^1^Department of Biomedical and Nutritional Sciences, University of Massachusetts, Lowell, MA, USA; ^2^Biomedical Engineering and Biotechnology Program, University of Massachusetts, Lowell, MA, USA; ^3^Department of Chemistry, University of Massachusetts, Lowell, MA, USA

## Abstract

**Introduction:**

Triglyceride (TG) is an independent risk factor for coronary heart disease. Previous work has shown that short-term supplementations of mouse chow with oxidized linoleic acid (OxLA) significantly reduce the level of plasma triglycerides.

**Study Objective:**

This study aims to determine the effects of longer-term supplementation of mouse chow with various concentrations of oxidized linoleic acid (OxLA) on plasma triglycerides.

**Study Design:**

The study consisted of forty C57BL/6 wildtype mice divided into four groups (*n* = 10). Two groups were kept as controls. One control group (P) was fed plain chow and the second control group (C) was fed chow supplemented with linoleic acid. The other two experimental groups (A) and (B) were fed oxidized linoleic acid supplemented chow in the following doses: 9 mg/day of oxidized linoleic acid and 18 mg/day of oxidized linoleic acid/mouse.

**Results and Conclusion:**

Mice that were on a diet supplemented with the higher dose of oxidized linoleic acid showed a 39% decrease in hepatic PPAR-*α* and a significant decrease in the plasma HDL levels compared to the mice that were fed diets of plain and linoleic acid supplemented chow. Interestingly, the longer-term consumption of oxidized linoleic acid may predispose to atheropathogenesis.

## 1. Introduction

Triglycerides plasma levels are major risk factor for cardiovascular diseases. It is a complex polygenic trait that is modulated by a number of major pathways in the intestine, liver, adipose, and plasma. Although triglycerides are not directly linked to the pathogenesis of atherosclerosis, they greatly influence plasma lipoproteins, especially the low-density and very low-density lipoproteins (LDL) which are known risk factors for cardiovascular disease (CVD).

Linoleic acid is the most abundant polyunsaturated fatty acid (PUFA) in many lipid rich diets. Like other polyunsaturated fatty acids (PUFA), linoleic acid is a ligand of the peroxisome proliferator-activated receptor alpha (PPAR-*α*) which is a regulating component of plasma triglyceride levels. We have previously shown that oxidized linoleic acid significantly lowers plasma triglyceride (TG) levels as compared to animals fed oleic acid. The changes were associated with increased APOA5 and acetyl-CoA oxidase genes expression in the mice that were fed a diet supplemented with 9 mg/mouse/day of OxLA. Two apolipoproteins (Apo), Apo A5 and Apo CIII, were of particular interest due to their role in the level of triglycerides. These proteins modulated triglycerides by interchangeably binding to VLDL particles [1, 2].

PPAR-*α* induces the expression of proteins involved in the uptake, transport, and metabolism of fatty acids which results in decreasing the synthesis of triglyceride. In human ApoA5 is a direct target of PPAR-*α* which is consistent with the triglyceride-lowering role proposed for ApoA5 [3]. However PPAR-*α* link to ApoA5 in mice is not well understood. ApoA5 induces a clearance of VLDL from the plasma through electrostatic interactions with heparin sulfate proteoglycan (HSPG). This interaction aid colocalizes the VLDL to HSPG attached lipoprotein lipase (LPL), allowing for greater lipolysis [1, 4–6].

ApoCIII, like ApoA5, localized on VLDL particles but has the opposite effect as it promotes increases of plasma triglycerides. Within the liver, ApoCIII acts by utilizing the available triglyceride substrates to increase synthesis of VLDL-TG. The increased synthesis of VLDL particles results in greater plasma TG levels. ApoCIII prevented lipolysis leading to increased plasma TG [6]. As a result, these two antagonistic proteins are considered key in triglyceride modulation [1, 4, 5].

Lipases are other proteins of interest in TG metabolism. They are key enzymes that break down lipids and lipoproteins; lipoprotein lipase (LPL) is antagonized by two angiopoietin-like proteins ANGPTL3/4. The proteins cause the LPL dimers to dissociate, leading to a loss of activity and lipolysis. ANGPTL3 is promoted when its gene is bound by the Liver X Receptor (LXR). Hepatic ANGPTL4 is activated by ligands of PPAR-*α*, which includes PUFA [3, 7].

By investigating the hepatic gene, protein expressions, and plasma protein levels, the study determines the range of modulators for plasma triglycerides, which is known to control biosynthesis and metabolism of TG and widely affects the plasma lipoproteins. The goal is to elucidate a mechanism by which oxidized linoleic acid modulates TG and hepatic lipoproteins metabolism.

## 2. Experimental Design

Normal C57BL/6 male mice were cared for in an animal facility with 12-hour light/dark cycles and all the protocols pertaining to this study were approved by the University of Massachusetts, Lowell, Institutional Animal Care and the Use Committee (IACUC.) The body masses of the mice were monitored over the course of 10 weeks. Water was provided ad libitum, while set amounts of diet were measured and supplied to each study group weekly.

## 3. Diets

13-Hydroperoxyoctadecadienoic acid (13-HPODE) was prepared as previously described [2]. Two prepared formulas of 13-HPODE were then shipped to Harlan Laboratories, Indianapolis, Indiana, US, for preparation of the experimental mouse chow formulas. The specialized diets were kept at 2°C until used. Four different diet formulas were provided to the groups of mice: a Standard Chow as plain control (P group), a chow supplemented with linoleic acid 9 mg/mouse/day, linoleic control (C group), oxidized linoleic acid, 9 mg/mouse/day (A group), and oxidized linoleic acid 18 mg/mouse/day diet (B group). Mice were fed the dietary formulas or kept on plain chow for two months.

## 4. Materials and Methods

Mice were euthanized at the end of the study. Whole blood was obtained from each mouse by heart puncture and placed in heparin tubes. The containers were spun in a cold centrifuge at 3000 rpm. The samples were later aliquoted and stored at −80°C. The plasma samples were analyzed for LDL, High-Density Lipoprotein (HDL), glucose, and total cholesterol using reagents and standards from Medica Corporation, Bedford, MA 01730. ApoA5, ApoCIII, ANGPTL3, ANGPTL4, and hepatic lipase (HL) in plasma were analyzed using commercial ELISA kits.

### 4.1. mRNA Extraction and Analysis of ApoA5, ApoCIII, PPAR-Alpha, and SREBP1 Genes

Prior to collection of organs, chilled 1x phosphate buffered saline (PBS) was perfused through the heart, after which the liver (100 mg samples) and adipose were collected in homogenization tubes. The organs were then flash frozen in liquid nitrogen (LN2) and stored at −80°C. Single aliquots of liver and adipose were stored in 1 mL of Trizol for later RNA extraction. The RNA was extracted from liver samples with the Trizol reagent and then aliquoted. RNA was quantified using the Qubit fluorimeter (Invitrogen, Thermo Fisher Scientific, Waltham, MA, USA). Quality was assessed on a 1% agarose gel using ethidium bromide as a probe and detected on a UVP imager (UVP Biosystems, Upland, CA, USA). cDNA was prepared using iScript RT mix, which was diluted to 1 : 50 for gene expression analysis. Aliquots of 8 *μ*L were run with a Polymerase Chain Reaction (PCR) Master mix containing EvaGreen SsoFast Supermix. ApoA5, ApoCIII, PPARa, and SREBP1 were run against GAPDH for control comparison.

### 4.2. Protein Extraction and Analysis of ApoCIII, ANGPTL3, and ANGPLT4

Hepatic protein was extracted through homogenization in 1 mL precipitation cocktail-10 mL radioimmunoprecipitation assay (RIPA) buffer with a complete mini ultra-protease inhibitor tablet. Homogenized samples were incubated on ice for 30 minutes and centrifuged for 15 minutes at 3000 rpm. The protein containing supernatant was removed and aliquoted. Concentrations were determined using the bicinchoninic acid (BCA) assay. All chemicals and materials used for the Western Blotting and gene expression were obtained from Bio-Rad Laboratories, Inc., Hercules, CA, USA.

For the western blot assay, 16 *μ*L of liver protein was loaded per lane on polyacrylamide 4–15% gels and ran with Western C Protein Plus Standards Ladder. The protein was then transferred onto 0.2 um PVDF membrane for blotting. A blocking buffer was prepared by dissolving nonfat dried milk (NFDM) in 1x Tris Buffered Saline (TBS) with  .1% v/v Tween 20 (T). The NFDM-TBST buffer was used to block membranes and dilute antibodies. ApoCIII, ANGPTL3, ANGPLT4, and proprotein convertase subtilisin/kexin type 9 (PCSK9) antibodies were assayed at 1 : 500. Their incubation was followed with a secondary goat anti-rabbit-HRP (1 : 10000) which was coincubated with an anti-ladder-HRP (1 : 10000). B-Actin, the reference protein, is HRP primary tagged (1 : 25000). All proteins were visualized on a UVP Biosystems Imager with Dura West ECL signaling reagent.

## 5. Results

### 5.1. Plasma Lipids, Glucose, and ELISA Measurements

**Figure 1:**
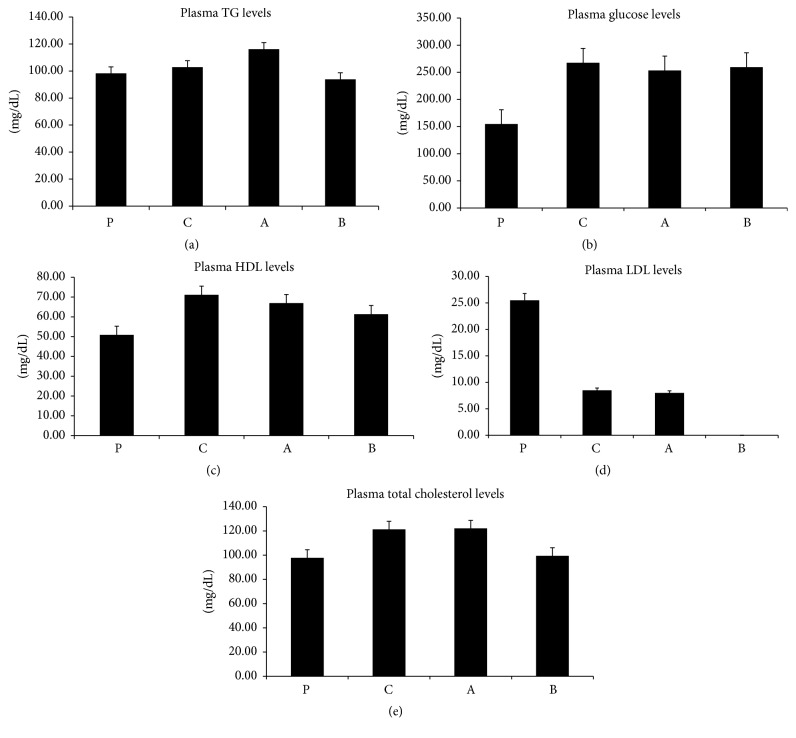
(a) The difference between the plasma levels of triglyceride in the 4 groups was not significant. (b) The difference between the plasma levels of glucose in plain control (Standard Chow) and control (chow supplemented with linoleic acid) is significant (*P* < 0.01); glucose also significantly (*P* < 0.01) decreased in higher concentration of OxLA compared to the plain chow. However the low oxidized LA has shown significantly (*P* < 0.01) increased glucose compared to the plain group. (c) Plasma HDL levels increased among all the treated groups; they were however more significant (*P* < 0.05) between the plain and control group and the LA and higher OxLA group (*P* < 0.01). (d) Higher OxLA plasma LDL levels were significantly (*P* < 0.01) reduced compared to the LA control. Plasma LDL was also greatly reduced in low OxLA compared to the plan and control groups. However the difference was not significant. (e) Total cholesterol plasma levels increased in LA and the low OxLA groups compared to the plain control and higher OxLA groups; however the difference was not significant.

The plasma was analyzed for lipids and glucose. Overall, the mice that were fed diets supplemented with fatty acids showed increasing plasma levels of glucose and lipids (Figures 1(a)–1(c) and 1(e)), with the exception of LDL (Figure 1(d)), which was decreased.

There were no substantial significant changes shown within the triglyceride measurements between the groups (Figure 1(a)) but a change in the weight gained by mice during the 10-week study was noted (data not included).

All groups that were fed a diet supplemented with fatty acids showed higher concentrations of total cholesterol (Figure 1(e)), glucose (Figure 1(b)), and HDL (Figure 1(c)) within their plasma.

Significantly greater blood glucose levels (Figure 1(b)) were seen in all three experimental groups. While the total cholesterol (Figure 1(e)) showed an overall increase, only the groups that were fed a diet supplemented with a lower concentration of the oxidized fatty acid showed much higher levels. HDL (Figure 1(c)) levels considerably increased in the linoleic acid fed control group compared to the plain control or high oxidized fed mice. The LDL (Figure 1(d)) levels were low in all groups and they were significant only when the high oxidized fed mice are compared to the linoleic control fed group.

#### 5.1.1. ELISA

**Figure 2:**
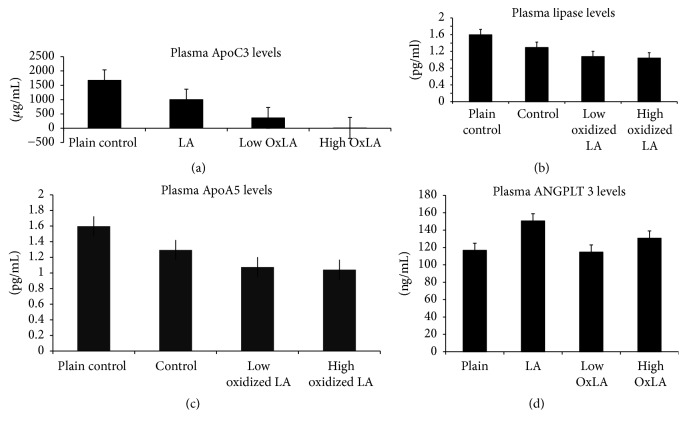
Plasma ApoC3 significantly decreased in a dose dependent manner in low OxLA (*P* < 0.05) compared to plain group. The levels were also significantly reduced (*P* < 0.05) between low OxLA and LA. Plasma ApoC3 levels were also significantly reduced (*P* < 0.01) in higher OxLA group compared to the LA. (b) Oxidized linoleic acid supplementation led to dose dependent significant (*P* < 0.05) decreases in plasma hepatic lipase when compared to the plain fed group of mice. The decreases in the hepatic lipase levels in the LA control group compared to the plain mice were not significant. (c) Plasma ApoA5 levels decreased but nonsignificantly in the treated groups compared to the plain control. (d) ANGPTL3 concentration decreased among the OxLA fed groups compared to the LA control linoleic acid group; interestingly the experimental groups had either similar or slightly elevated ANGPTL3 concentration compared to the plain groups.

Linoleic acid or oxidized linoleic acid supplementation has led to a decrease in plasma ApoCIII (Figure 2(a)) levels. The drop was significant for both of the oxidized groups compared to the linoleic acid control group. The greatest drop was in group (B) which had the highest concentration of oxidized linoleic acid, demonstrating a dose dependent response. 

*Plasma Hepatic Lipase (HL)*. Plasma HL decreased within experimental groups (Figure 2(b)). The drop was significant for the OxLA groups compared to the plain groups; these differences were dose dependent.

*Plasma ApoA5 and ANGPTL3*. The group of mice that were fed the linoleic acid and 18 mg/mouse/day oxidized linoleic acid supplemented chows showed increased plasma levels of ApoA5 (Figure 2(c)) and ANGPTL3 (Figure 2(d)). The difference was not significant. The ApoA5 levels were elevated in the high oxidized linoleic acid group. However, ANGPTL3 had the highest concentration in the control linoleic acid group.

### 5.2. Gene Expression

**Figure 3:**
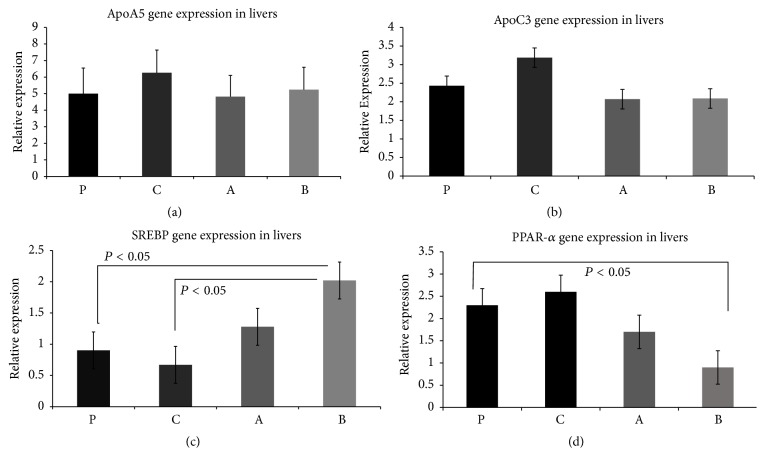
(a) ApoA5 is significantly upregulated in the linoleic acid control group (C group). P group (Standard Chow), C group (chow supplemented with linoleic acid 9 mg/mouse/day), A group (chow supplemented with oxidized linoleic acid 9 mg/mouse/day), and B group (chow supplemented with linoleic acid 18 mg/mouse/day). (b) ApoC3 is nonsignificantly upregulated in the linoleic acid control group (C group). P group (Standard Chow), C group, A group (chow supplemented with oxidized linoleic acid 9 mg/mouse/day), and B group (chow supplemented with linoleic acid 18 mg/mouse/day). (c) SREBP gene expression shows significant upregulation in the mice group fed a high concentration of oxidized linoleic acid. P group (Standard Chow), C group (chow supplemented with linoleic acid 9 mg/mouse/day), A group (chow supplemented with oxidized linoleic acid 9 mg/mouse/day), and B group (chow supplemented with linoleic acid 18 mg/mouse/day). (d) Slight peak in the PPAR-*α* gene expression in the linoleic acid control group (C group) and decreased expression in both oxidized linoleic acid concentration groups (A and B groups): P group (Standard Chow), C group (chow supplemented with linoleic acid 9 mg/mouse/day), A group (chow supplemented with oxidized linoleic acid 9 mg/mouse/day), and B group (chow supplemented with linoleic acid 18 mg/mouse/day).

ApoA5 gene was significantly upregulated (Figure 3(a)) in the linoleic acid control group. This aligns with the result for the plasma APOA5 measured by ELISA. The increase in the expression of ApoCIII noted in the linoleic acid control group was not significant (Figure 3(b)). SREBP gene expression was slightly downregulated in the linoleic acid control group. However, it was upregulated (Figure 3(c)) in the oxidized groups. The upregulation was significant for the mice that were fed high OxLA diet.

PPAR-*α* expression peaked slightly for the linoleic control group and dropped for both oxidized linoleic acid groups (Figure 3(d)).

### 5.3. Western Blot

**Figure 4:**
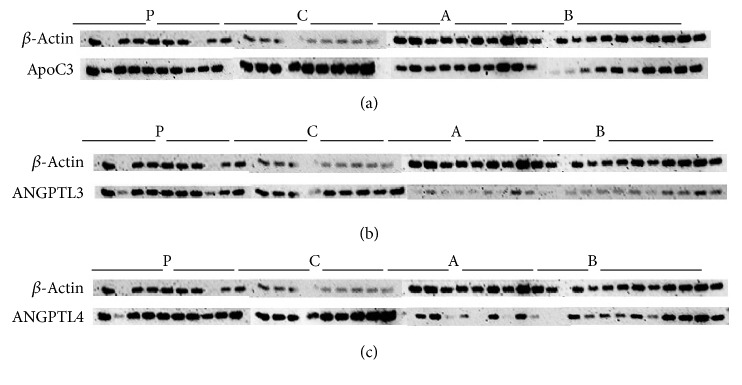
(a) Increased expression of ApoC3 over the contrast B-actin in all groups with more intense expression in group C while the B group seems unchanged. P group (Standard Chow), C group (chow supplemented with linoleic acid 9 mg/mouse/day), A group (chow supplemented with oxidized linoleic acid 9 mg/mouse/day), and B group (chow supplemented with linoleic acid 18 mg/mouse/day). (b) ANGPTL3 protein expression is increased in groups P and C, while it is underexpressed in groups A and B, with group showing much less expression. P group (Standard Chow), C group (chow supplemented with linoleic acid 9 mg/mouse/day), A group (chow supplemented with oxidized linoleic acid 9 mg/mouse/day), and B group (chow supplemented with linoleic acid 18 mg/mouse/day). (c) ANGPTL4 protein expression is increased intensely in group C, as well as the P group, which also shows a slight increase. Group A shows no change in expression levels while the B group displays much less expression between the dose dependent groups (A and B). P group (Standard Chow), C group (chow supplemented with linoleic acid 9 mg/mouse/day), A group (chow supplemented with oxidized linoleic acid 9 mg/mouse/day), and B group (chow supplemented with linoleic acid 18 mg/mouse/day).

ApoCIII (Figure 4(a)) shows greater expression for the ApoCIII protein for all groups except the B group, which appears unchanged. The C group showed the highest protein expression followed by the P group and A group with the least upregulation.

ANGPTL3 (Figure 4(b)) shows intense protein expression for the P and C groups. The A group showed significant underexpression and the B group showed lesser expression.

ANGPTL4 (Figure 4(c)) showed significant ANGPTL4 overexpression on the linoleic acid control group. There was a slight increase in expression for the plain group. The high oxidized linoleic acid group showed no change and the low oxidized linoleic acid group showed differential expression between samples.

## 6. Discussion

Linoleic acid (LA) is an essential fatty acid that is required for physiological and developmental functions of mammalians, particularly humans. Like all polyunsaturated fatty acids (PUFAs), LA is susceptible to oxidation that results in several active metabolites that have biological relevance. 13-Hydroxyoctadecadienoic acid (13-HODE) (a common name for 13(S)-hydroxy-9Z, 11E-octadecadienoic acid (13(S)-HODE)) and 9-hydroxyoctadecadienoic acid (9-hydroxy-10(E),12(Z)-octadecadienoic acid or 9-HOPDE) are the most studied metabolites of LA. In the current study we used 9-HOPDE derivative to reassess our previous findings that showed significant reduction on TG after over two weeks of 9-HOPDE dietary intake. The present results of our study showed differential responses for how oxidized linoleic acid affects triglyceride metabolism in C57BL/6 mice. It appears that the prolonged dietary intake of the oxidized linoleic acid has a different effect than what we previously reported [2] on the short intake acute effect on TG and lipoprotein metabolism. This may suggest that extended dietary intake of oxidized fatty acids results in mixed favorable and nonfavorable liver and plasma responses. We have seen some genes and plasma lipoproteins levels changes that are dose dependent for intake of oxidized linoleic. However, it is not evident that the intake of dietary OxLA resulted in significant metabolic alterations in TG and lipoprotein.

The plasma triglyceride levels were the highest for the group that had less oxidized linoleic acid in their diet which in part incorporates our previous findings. Interestingly, plasma triglyceride levels are comparable to the gain in body weight over the 10-week period (data not shown). ApoA5 is an important lipid modulating protein that acts on TG and VLDL particles. ApoA5 itself is a protein that is not highly expressed. It is described to stabilize lipid droplets in the liver and protects LPL through an electrostatic mechanism in the bloodstream. The relative abundance of mRNA (Figure 3(a)) for ApoA5 in the liver was low, although it did have a significantly higher concentration in the linoleic acid control group. There was a lower manifestation in the low oxidized group. In comparison, the plasma level showed no significant differences for ApoA5 (Figure 2(c)). However, it demonstrated greater concentration in the bloodstream for the high oxidized and control groups compared to the low oxidized group. ApoCIII is a hepatic and plasma protein that acts to block LPL activity and causes an increase in TG levels in the blood stream. Gene expression (Figure 3(b)) data showed a greater, though insignificant, abundance of mRNA for ApoCIII in the livers of the control linoleic acid group, while there was a lower abundance seen in the mice fed oxidized linoleic acid. The western blot (Figure 4(a)) data generally agreed with the gene expression. The highest amount of active hepatic protein was found in samples from the linoleic control group followed by the plain control group. The low oxidized group showed higher expression than the higher oxidized group. Plasma (Figure 2(a)) ApoCIII concentration was lower in the groups fed fatty acids diets. However, it was more significantly lower in those fed oxidized diets. This could possibly be due to cellular degrading of ApoCIII during or after translation, or the protein may have a loss of function in these mice. Plasma glucose (Figure 1(b)), HDL (Figure 1(c)), and total cholesterol (Figure 1(e)) had some differential changes across mice samples. The glucose increased in all the experimental groups, while the plasma total cholesterol was higher in the linoleic acid control and lower oxidized linoleic acids fed groups compared to the mice on plain chow and high oxidized linoleic acids fed groups. SREBP is known to modulate the regulation of ANGPTL3, LDLR, and PCSK9, all of which affect the plasma lipids profile [8, 9]. SREBP may have induced more expression of the LDL receptors leading to the much lower LDL concentrations (Figure 1(d)) [9].

SREBP is equally produced in the intestine and liver. It also has a secondary function in increasing triglyceride rich lipoprotein production within the intestine. By promoting the activity of MTP, PCSK9 increases lipidation of ApoB that can lead to greater plasma lipid concentrations [9]. SREBP increases activities of ANGPTL3 and SREBP also with ANGPTL4 antagonized LPL and HL which affect their activity [6, 7]. The ANGPTL family of proteins causes dissociation of many lipases. The dissociation causes a loss of activity and decreased clearance of plasma triglycerides, which is one of the reasons why VLDL particles have less lipolysis thus leading to a diminished clearance. ANGPTL3 and ANGPLT4 are essential for LPL regulation [10]. ANGPTL3 (Figure 4(b)) expression levels were regulated for P and C groups, while group A has shown significant reduction and group B slight reduction. ANGPTL3 was upregulated in group C, though not significant, in the plasma (Figure 2(d)). Although ANGPTL3 did not have any significant changes, it may have acted in preventing further clearance of triglycerides [11].

ANGPTL4 has shown significant upregulation in the linoleic control group as shown in the western blot (Figure 4(c)).

*Conclusion*. This study demonstrates the ambiguity of the prolonged dietary oxidized fatty acids intake. The rationale for the differences between the short and extended period intake of the oxidized linoleic fatty acid and the conflicting outcomes compared to our previous study is not very clear. However, apparently the long term intake of oxidized linoleic acid may have unfavorable effects on lipoprotein metabolism. The mechanisms of actions vary greatly between the experimental formulas and controls. These findings strongly point towards the proatherogenic roles of the oxidized fatty acids. Future studies using LDLr −/− mouse models may be necessary to establish possible linkages to the pathogenesis of atherosclerosis [12, 13].
